# Penetrance interactions of colour pattern loci in the African Monarch and their implications for the evolution of dominance

**DOI:** 10.1002/ece3.11024

**Published:** 2024-02-27

**Authors:** Richard H. ffrench‐Constant, Jonathan Bennie, Ian J. Gordon, Lorna Depew, David A. S. Smith

**Affiliations:** ^1^ Centre for Ecology and Conservation University of Exeter Penryn UK; ^2^ Centre for Geography and Environmental Science University of Exeter Penryn UK; ^3^ Department of Biology, College of Science and Technology University of Rwanda Kigali Rwanda; ^4^ ICIPE Nairobi Kenya; ^5^ Natural History Museum Eton College Windsor UK

**Keywords:** admixture polymorphism, African Monarch, *Danaus chrysippus*, expressivity, heterozygote, mimicry, penetrance

## Abstract

Scoring the penetrance of heterozygotes in complex phenotypes, like colour pattern, is difficult and complicates the analysis of systems in which dominance is incomplete or evolving. The African Monarch (*Danaus chrysippus*) represents an example where colour pattern heterozygotes, formed in the contact zone between the different subspecies, show such intermediate dominance. Colour pattern in this aposematic butterfly is controlled by three loci *A*, *B* and *C*. The *B* and *C* loci are closely linked in a *B*/*C* supergene and significant interaction of *B* and *C* phenotypes is therefore expected via linkage alone. The *A* locus, however, is not linked to *B*/*C* and is found on a different chromosome. To study interactions between these loci we generated colour pattern heterozygotes by crossing males and females bearing different *A* and *B*/*C* genotypes, collected from different parts of Africa. We derived a novel scoring system for the expressivity of the heterozygotes and, as predicted, we found significant interactions between the genotypes of the closely linked *B* and *C* loci. Surprisingly, however, we also found highly significant interactions between *C* and the unlinked *A* locus, modifications that generally increased the resemblance of heterozygotes to homozygous ancestors. In contrast, we found no difference in the penetrance of any of the corresponding heterozygotes from crosses conducted either in allopatry or sympatry, in reciprocal crosses of males and females, or in the presence or absence of endosymbiont mediated male‐killing or its associated *neoW* mediated sex‐linkage of colour pattern. Together, this data supports the idea that the different colour morphs of the African Monarch meet transiently in the East African contact zone and that genetic modifiers act to mask inappropriate expression of colour patterns in the incorrect environments.

## Graphical abstract

The African Monarch or African Queen shows intermediate dominance in the colour pattern heterozygotes formed across the broad East African contact zone. Here we quantify the level of penetrance of each heterozygote formed by interactions between the *A*, *B* and *C* colour pattern loci. We find that the *A* locus interacts with the *B*/*C* supergene and is thus an example of a genetic modifier.

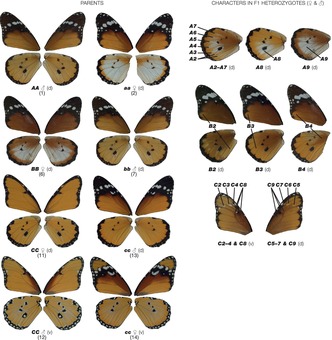

## 1 INTRODUCTION

The evolution of dominance in complex traits has been extensively debated in the history of evolutionary genetics (Bagheri, 2006). Dominance can be defined as the relative effects on the overall phenotype of the two different gene alleles found at any given locus, with dominant alleles inferred to be more active at the molecular level and recessive alleles less so (Kacser & Burns, 1981; Wilkie, 1994). In turn, intermediate dominance is used to refer to a heterozygous phenotype intermediate between that of two homozygous parents. However, given that dominance is often an emerging trait in a population, as a new favoured dominant allele will increase rapidly, the exact levels of dominance shown by any individual heterozygote for any given locus will differ both with genetic background and how recently the trait has appeared (Bourguet et al., 1997). Specifically, variations in dominance can be controlled by the presence of *trans*‐acting factors encoded at a distance to the locus of interest, often termed as genetic modifiers (Bourguet et al., 1997). The role of these modifiers is often predicted but well‐documented examples remain rare (Sheppard et al., 1985; Timmermans et al., 2014). Heterozygotes displaying different levels of phenotypic expression can in turn be defined as varying in their expressivity at the level of the individual. Overall penetrance can then be measured as the proportion of individual heterozygotes in a genetic cross, or population, expressing any given intermediate phenotype or level of character expressivity. However, given that the intermediate phenotypes shown by complex heterozygotes can be hard to classify, a rigid and reproducible classification of the expressivity of each intermediate form is necessary for further studies.

Supergenes controlling butterfly mimicry (Joron et al., 2011; Kunte et al., 2014; Timmermans et al., 2014) are an excellent system in which to study the evolution of dominance as abundant warning patterns should be avoided by predators (Smith, 1979). In populations showing colour pattern polymorphism controlled by such supergenes, intermediate forms should be more readily predated and correspondingly less fit in a population context. In turn, this should select against heterozygotes with low levels of penetrance as they are more readily discernible as being intermediate by any given predator. Critically, however, these predictions differ in systems where dominance is evolving in sympatry, where colour pattern alleles frequently co‐occur in the same population, or in allopatry, where the two alleles of any given heterozygote are less likely to be found together in nature. This prediction is well illustrated by two classic examples of butterfly mimicry, the longwing butterfly *Heliconius numata* and the swallowtail butterfly *Papilio dardanus*. Thus, crosses conducted within a geographic morph of *P. dardanus* show stronger dominance than crosses between different geographic morphs taken from different parts of Africa (Clarke & Sheppard, 1960a; Nijhout, 2003). Correspondingly, crosses between different melanic morphs of *H. numata*, controlled by the *P* for *Pattern* supergene, show strong levels of dominance as they all fly together in sympatry (Le Poul et al., 2014). Finally, it is worth noting that many of the expectations of hybrid zone behaviour come from contact zones where only two morphs meet, the more complicated example discussed here of four morphs coming together across a broad region therefore raises further unanswered questions.

The African Monarch represents an unusual mimicry system in which different colour morphs are found as largely monomorphic populations in different parts of Africa (Liu et al., 2022). Colour pattern is controlled by three major loci termed *A*, *B* and *C*, with *B* and *C* being closely linked on the same chromosome as the *B*/*C* supergene. The *A* locus controls the extent of the white hindwing patch, a phenotype found predominantly in the West of Africa (Liu et al., 2022), and the *B*/*C* supergene controls the pattern and extent of both black and white elements on the butterfly forewing (Smith et al., 1998). The different colour morphs converge in a broad contact zone across East Africa, where they are thought to be brought together by the winds surrounding the movement of the Inter Tropical Convergence Zone or ITZC (Smith et al., 1997). The precise extent of the contact zone between the different morphs has been recently described in detail using extensive citizen science data and we refer the reader to the distributions described by Liu et al. (2022). We view this recent meeting of the different morphs as an example of admixture polymorphism (Smith et al., 2021; Wall, 2000), as the morphs can readily interbreed, and the corresponding heterozygotes are therefore common across the contact zone (Smith et al., 1997). The African Monarch therefore differs from other classic examples of colour polymorphism in that colour pattern alleles in the corresponding heterozygotes meet transiently and their expected fitness in the face of predation remains unclear. The population genetics of this polymorphic contact zone is further complicated by the emergence of a recent infection by a single strain of a male‐killing mollicute, *Spiroplasma*, that appears to have infected a single *neoW* carrying (*B*/*C* supergene carrying autosome 15 fused to the female linked *W* chromosome) matri‐lineal line of butterflies (Smith et al., 2019), forming what we refer to here as the *Spiroplasma‐neoW* complex. The B/C supergene has arisen in an interesting stepwise fashion and we refer the reader elsewhere for a discussion of the molecular origins of the colour pattern supergene (Kim et al., 2022).

**FIGURE 1:**
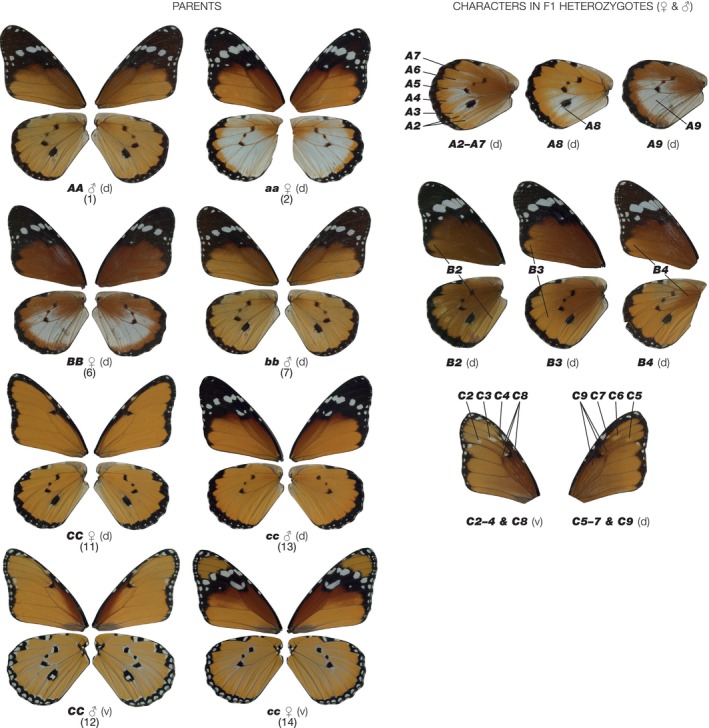
Scoring system for expressivity of colour patterns in heterozygotes of the African Monarch butterfly. d = dorsal, v = ventral side of butterfly. Nomenclature of spaces and veins follows the Comstock system. Crosses *AA* (1) × *aa* (2), *BB* (6) × *bb* (7), *CC* (11–12) × *cc* (13–14). F1 characters: *A* locus: (3) white scales along veins 1 + 2A and 3A (A2), Cu1b (A3), Cu1a (A4), M3 (A5), M2 (A6), M1 (A7); (4) small white patch in cell and in spaces Cu1b‐M3 (A8); (5) large white patch in cell and spaces 1 + 2A, 3A and inner margin (A9). B locus: (8) B2, forewing mainly brown with marginal orange in spaces 1 + 2A and Cu1b, hindwing largely brown with orange confined to distal margin; (9) B3, forewing as B2, hindwing brown proximally and orange distally; (9) B3, forewing mainly brown (as B2), hindwing brown proximally and orange distally; (10) B4, forewing mainly brown (as B2), hindwing orange with brown pigment confined to cell. C locus: white (pale) spot on ventral side of space M3 (C2), M2 (C3), M1 (C4), on dorsal side of space M3 (C5), M2 (C6), M1 (C7); black scales in any of the ventral (15) or dorsal (16) surfaces of spaces M1–M3 and the cell. Expressivity scores (ES) for heterozygotes. A locus, 0–8: complete dominance of *A* = 0. ES points are one point for each of A2–7, 7 for A8 and 8 for A9. B locus, 0–3: complete dominance of *B* = 0. B2 = ES1, B3 = ES2, B4 = ES3. C locus, 0–8: complete dominance of *C* = 0. C2–C9 score one ES point each.

As a first step in studying the likely fitness of African Monarch heterozygotes in the contact zone, we wanted to first define the level of penetrance shown in crosses between parents of differing geographic location which carry different combinations of the *A* and *B*/*C* supergene loci. Here we define an expressivity scale for each of the different heterozygotes by scoring the presence‐absence of colour in different wing compartments. This new scoring system uses both variation in colour and spatial pattern elements by scoring colours within the well‐documented venation compartments of butterflies and moths (see caption of Figure 1 for details). By scoring the corresponding African Monarch heterozygotes in reciprocal crosses and by using parents collected from either sympatric or allopatric populations, we can then score the penetrance of each heterozygote in each parental cross. Because the *B* and *C* loci are found on the same chromosome and are locked together in a chromosomal inversion, as the *B*/*C* supergene, we should expect interactions due to their close linkage. In contrast, the *A* locus is encoded on a different chromosome and interactions with *B*/*C* are therefore unexpected. Here, consistent with the recent and ongoing mixing of these subspecies in the contact zone, we find no effect of allopatry, sympatry, sex, *Spiroplasma* infection status, or the corresponding colour pattern sex‐linkage, on the penetrance of the corresponding colour pattern heterozygotes at any of the three loci. We do however see strong interactions between all three of the colour pattern loci themselves. The implications of these findings for the potential emergence of dominance in this unique case of admixture polymorphism are discussed.

## 2 MATERIALS AND METHODS

### 2.1 Source and infection status of parents

In order to test the effects of sympatry and allopatry on the penetrance of heterozygotes we selected parents from a range of different geographical sources, that are known to show different alleles at each of the *A* and *B*/*C* loci. Parent butterflies came from, Cape Coast, Ghana (mainly the genotypes *aa* *bc* / *bc* ); Nairobi, Kenya (mainly *AA* *bC* / *bC* ); Dar es Salaam, Tanzania (*AA* *bC* / *bC* and *AA* *Bc* / *Bc* ) and Harare, Zimbabwe (only *AA* *Bc* / *Bc* ) (see raw data table on FigShare). To derive adult males and virgin females for each cross, eggs were collected from the field and raised to adulthood at a single location in Harare, Zimbabwe. To look at the potential effects of the sex of the parents on resulting heterozygote penetrance reciprocal crosses of male × female and female × male for any given colour pattern were carried out. Further, the sex of the corresponding progeny was also scored, as well as the inferred presence‐absence of the endosymbiotic mollicute *Spiroplasma*, and the corresponding presence‐absence of its associated male‐killing (defined as all female broods where all the males have been killed by *Spiroplasma* infection) and sex‐linked colour pattern (*neoW* associated) phenotypes. In the results, below, we therefore provide data for male‐killing and sex‐linkage separately (see Tables 1, 2, 3), as some broods still show sex‐linkage but appear to have secondarily lost the male‐killing *Spiroplasma* by self‐curing, presumably associated with failed transmission of the male‐killing molicute.

### 2.2 Scoring of colour pattern heterozygotes

For each of the colour pattern crosses we scored the heterozygous colour patterns using a novel expressivity scoring system (see Figure 1 for details). For the *A* locus this scale defines the extent of the white hindwing patch in reference to defined veins and wing compartments, using a nine‐point scale of A1–A9. For the *B* locus the scale defines the extent of brown colouration on the wing using a four‐point scale of B1–B4. Finally, for the *C* locus the extent of the black and white pattern elements across the forewing is defined by a nine‐point scale defined as C1–C9 (Figure 1). The penetrance of each character in each of the corresponding heterozygotes was defined as the frequency in any given cross, or groups of similar crosses, and results are therefore displayed as histograms of the frequency of each character state in each group of the resultant progeny. Statistical analysis was performed separately for all data sets with each locus being treated separately. Analysis was achieved by modelling the total penetrance score on each locus with a linear mixed‐effects model, with parental genotype at the locus, genotype at the other loci, sex, male‐killing and sex‐linkage as fixed effects brood as a random effect. Linear mixed effects models were built using the nlme package (v4.1.2) in R (Pinheiro et al., 2021).

## 3 RESULTS

### 3.1 Interactions between the colour pattern loci

**FIGURE 2:**
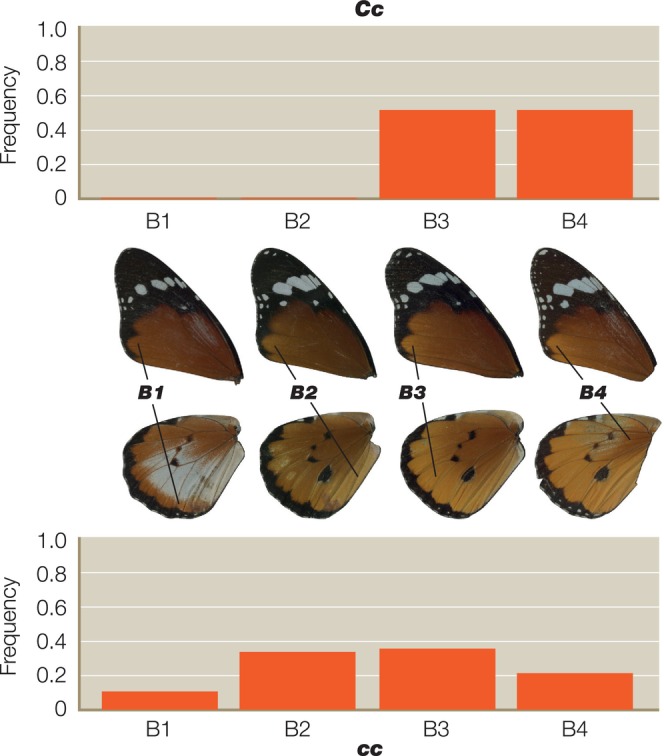
Penetrance histograms showing the frequency of each expressivity score for the *B* locus and its significant interaction with the *C* locus (see accompanying Table 1 for statistics). Histograms are the frequency of individual scores for the expressivity characters B1–4 in animals that differ in their inferred genotype for the *C* locus (*Cc* above and *cc* below) where the *B* genotype is *Bb* throughout. Total numbers scored represent 145 heterozygotes from each of eight different broods.

**FIGURE 3:**
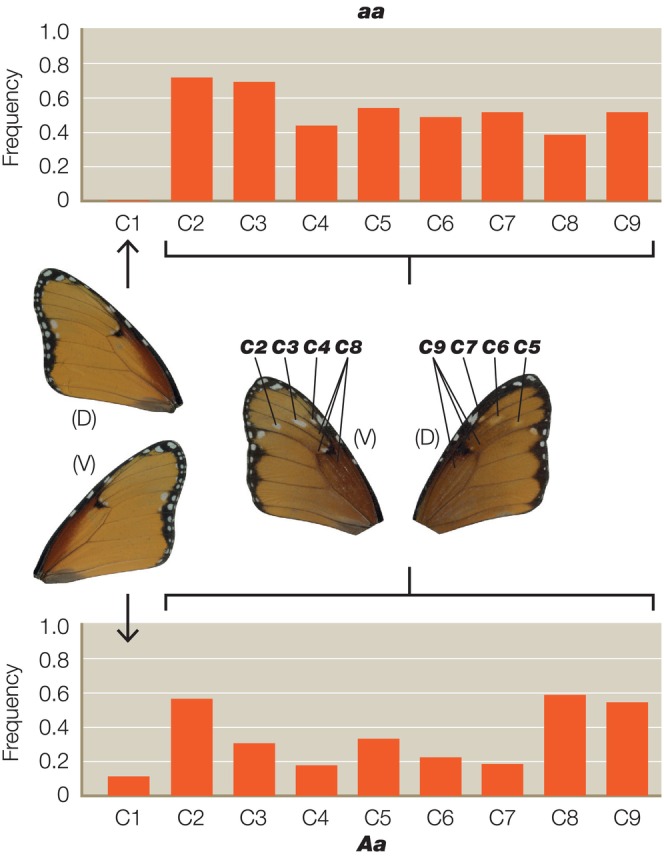
Penetrance histograms showing the frequency of each expressivity score for the *C* locus and its significant interaction with the *A* locus (see also Table 2). Histograms are the frequency of individual scores for the expressivity characters C1–9 in animals that differ in their inferred genotype for the *A* locus (*aa* above and *Aa* below) where the *C* genotype is *Cc* throughout. Total numbers scored represent 148 heterozygotes from 12 different broods.

**FIGURE 4:**
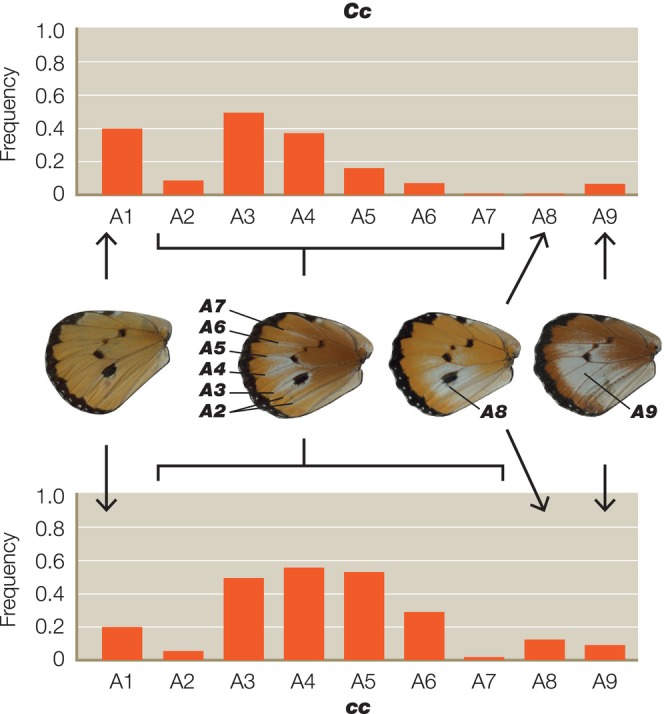
Penetrance histograms showing the frequency of each expressivity score for the *A* locus and its significant interaction with the *C* locus (see also Table 3). Histograms are the frequency of individual scores for the expressivity characters A1–9 in animals that differ in their inferred genotype for the *C* locus (*Cc* above and *cc* below) where the *A* genotype is *Aa* throughout. Total numbers scored represent 368 heterozygotes scored from 21 different broods.

We scored a total of 661 individual heterozygotes from 41 different broods, representing 368 butterflies from 21 broods for the A locus, 145 animals from eight broods for the B locus and 148 animals from 12 broods for the C locus. As predicted, we found significant interactions between the genotypes of the closely linked B and C loci (Table 1). The *C* allele increases the penetrance of *b* in *Bb* butterflies, while *c* reduces it (Figure 2). Surprisingly, however, we also found significant interactions between *C* and the unlinked A locus (Tables 2 and 3), with the interaction between the *C* and *A* alleles being more highly significant (*p* < .0004) (Table 2) than the reciprocal interaction between *A* and *C* (*p* < .0282) (Table 3). The *A* allele therefore increases the penetrance of *c* in *Cc* butterflies, whereas *a* reduces it (Figure 3). However, the modifier effect of both *A* and *a* in *Cc* butterflies is bimodal (Figure 3), suggesting that there might in fact be two distinct *c* alleles (see Section 4). In the reciprocal A–C interaction (Figure 4), *c* increases penetrance of *a* in *Aa* butterflies, whereas *C* reduces it.

**TABLE 1:** Interactions between the *B* locus and the *A* and *C* colour pattern loci for parents of different origin, sex and *Spiroplasma* infection status.

	df	*F*‐value	*p*‐Value
Origin of parent	3	0.78463	.5046
Sex	1	0.92261	.3386
*A* locus interaction	2	1.36343	.2594
***C* locus interaction**	1	6.70671	**.0107**
Male‐killing	1	0.30693	.6034
Sex‐linkage	1	0.44629	.5337

*Note*: Broods were scored for the presence of male‐killing and/or the presence of the anticipated *Spiroplasma‐neoW* associated sex‐linkage of colour pattern. Note that only the interaction with the *C* locus is significant at the 5 percent level as highlighted in bold. See Figure 2 for accompanying penetrance histograms. The number of degrees of freedom (df), *F*‐value and *p*‐value are shown for each variable.

**TABLE 2:** Interactions between the *C* locus and the *A* and *B* colour pattern loci for parents of different origin, sex and *Spiroplasma* infection status.

	df	*F*‐value	*p*‐Value
Origin of parent	3	0.32187	.8095
Sex	1	1.05154	.3071
***A* locus interaction**	1	13.39238	**.0004**
*B* locus interaction	1	0.17666	.6750
Male‐killing	1	0.53806	.4801
Sex‐linkage	1	1.19080	.2772

*Note*: Note the highly significant interaction between the *C* and *A* locus, highlighted in bold. See Figure 3 for accompanying penetrance histograms. For key to the other variables scored see caption for Table 1.

**TABLE 3:** Interactions between the *A* locus and the *B* and *C* colour pattern loci for parents of different origin, sex and *Spiroplasma* infection status.

	df	*F*‐value	*p*‐Value
Origin of parent	4	1.20088	.3102
Sex	4	1.41022	.2359
*B* locus interaction	1	0.00355	.9525
***C* locus interaction**	1	4.85792	**.0282**
Male‐killing	1	2.00557	.1729
Sex‐linkage	1	1.16471	.2813

*Note*: Note the significant interaction between the *A* and *C* locus, highlighted in bold. See Figure 4 for accompanying penetrance histograms. For key to the other variables scored see caption for Table 1.

### 3.2 Parental origin and infection status

In contrast to the interaction between the loci themselves, we found no statistically significant effects for either the origin or sex of the parents, or the sex of their progeny, for any of the crosses. We also found no significant effect on colour pattern penetrance exerted either by male‐killing (as scored by the presence of all female broods) associated with *Spiroplasma* infection or the associated *neoW* sex‐linked colour pattern inheritance (involving a fusion of the *B*/*C* carrying chromosome 15 with the female linked *W* chromosome; Martin et al., 2020) shown in most of the mollicute infected broods (see bottom two rows in each of Tables 1, 2, 3). Note that some broods still showed *neoW* associated sex‐linkage but had secondarily lost the male‐killing endosymbiont and are therefore self‐cured of the parasite, thus necessitating the scoring of both male‐killing and sex‐linked colour pattern as independent variables.

## 4 DISCUSSION

The African Monarch shows unexpected polymorphism in colour pattern across a broad contact zone which extends across much of East Africa (Smith et al., 1997, 1993). This contact zone represents the meeting of the different colour pattern morphs that are largely monomorphic in their source populations but can freely interbreed when found together (Liu et al., 2022). This situation contrasts markedly with the well‐studied example of melanic morphs of *H. numata*, controlled by the *P* supergene, which shows high levels of dominance and where all the morphs fly together in sympatry (Bastide et al., 2023). In the African Monarch, however, the transient nature of the admixture polymorphism, which is thought to be driven by the winds surrounding the movement of the ITCZ across East Africa (Smith & Owen, 1997), would be expected to afford little chance for the evolution of dominance in the associated colour pattern alleles. This is because each of the African Monarch source populations represents highly monomorphic subspecies, or colour morphs, and the different colour pattern alleles at each locus are not normally found flying together (Liu et al., 2022). As colour pattern is controlled by only three major loci, *A*, *B* and *C*, and both *B* and *C* are combined in a *B*/*C* supergene closely linked on one chromosome, this represents an ideal system in which to examine the dominance evolution of the *B*/*C* supergene itself and any potential interaction with the distantly encoded *A* locus as a potential genetic modifier. In this study we have therefore asked three simple questions. First, is there any effect on heterozygote penetrance in crosses conducted in sympatry or allopatry? Second, is there any evidence for interactions between the colour pattern loci themselves? Third, and finally, are such interactions affected by the sex of the parents or their infection status with the male‐killing mollicute *Spiroplasma*, which is also found together with a sex‐linked version of *B*/*C* carried on a *neoW* chromosomal fusion?

Here we have shown that *B* and *C* do indeed interact as predicted by their proximity in the genome, where they are effectively locked together within an inversion which would be expected to suppress recombination between *B* and *C* (Kim et al., 2022). In effect, *C* modifies *Bb* phenotypes towards orange, whereas the recessive *c* allele renders them browner. In so doing, the *Bc* / *bC* heterozygote comes to more closely resemble the *bC* / *bC* phenotype found in the subspecies *klugii*, the predominant morph in the North‐east of Africa. In contrast, in making the *Bc* / *bC* phenotype browner, it comes to more closely resemble the *Bc* / *Bc* phenotype found in subspecies *orientis*, the predominant morph in southern Africa. We note, however, that these clear‐cut results from the *B* locus are taken from a limited number of individual broods derived from individual laboratory‐based crosses. With the potential for other modifiers of the brown colour encoded by the *B* locus in the field, we cannot therefore guarantee that the dominant brown homozygous phenotype or the corresponding heterozygotes are strictly comparable between broods. Surveys of larger numbers of field‐collected butterflies are therefore clearly needed for further examination of the genetic basis of brown colouration. Intriguingly, we also note that despite the physical linkage and close genetic proximity of the *B* and *C* locus within the supergene that one of the six sequenced haplotypes (Kim et al., 2022) is indeed a recombinant. Recombination within supergene is unexpected and may further complicate the penetrance of colour patterns derived from the *B*/*C* supergene.

Surprisingly, however, we do also see significant interactions between the *A* and *C* loci, despite the *A* locus being on a completely different chromosome to *B*/*C*. In detail, the recessive allele of *a* increases penetrance of *c* in *Cc* butterflies, and in so doing improves their resemblance to the *aa* *bc* / *bc* genotype found in subspecies *alcippus*, the dominant morph in West Africa. To reinforce this effect, the recessive allele *c* increases the penetrance of *a* in *Aa* butterflies, whereas *C* reduces white in the hindwing of this phenotype and, in so doing, improves its resemblance to the orange hindwing of *AA* *bC* / *bC* found in subspecies *klugii*. Thus, the *A* locus is a modifier of the *B*/*C* supergene phenotype, and vice versa. Interestingly, the modifier effects of both *A* and *a* in *Cc* butterflies is bimodal (Figure 3), suggesting the possibility that there might be two distinct *c* alleles, one in the South African morph *orientis* (*AA* *Bc* / *Bc* ) and the other in West African *alcippus* (*aa* *bc* / *bc* ), which are modified in opposite directions at the *A* locus. We note that such phenotypic differences between the two geographical *cc* forms were first noted by Pierre (1973).

In terms of likely mechanisms of colour pattern pigmentation, as both the *A* and *C* loci encode white pattern elements it seems likely that the *A* locus either facilitates the expression of white in specific regions of both the fore‐ and hindwing or that the gene product of the *A* locus is somehow involved in the balance (pigment production or pigment transport) between orange‐white pigmentation. Whilst a homologue of the *Drosophila yellow* gene, known to encode a carrier protein involved in melanin deposition (Wittkopp et al., 2002), is encoded at the *B* locus (Martin et al., 2020), the genes encoded at either *A* (unknown at present) or *C* (potentially a homologue of the *Drosophila* gene *Arrow*; Martin et al., 2020), however, remain less clear. Whilst the evolution of supergene modifiers has been discussed extensively in the literature on the evolution of butterfly colour pattern supergenes (Clarke & Sheppard, 1972, 1975, 1959, 1960b, 1960a, 1962), the documented evidence for such loci is still rare and the demonstrated interaction of the distant *A* locus with the B/C supergene is therefore noteworthy.

Aside from the significant penetrance interactions between heterozygotes of the *A*, *B* and *C* loci, we saw no significant effects of either the sex of the parents or their progeny, the *Spiroplasma* infection status, or the origin of the parents used in any of the different colour pattern crosses. These observations are consistent with inferences from recent molecular data which suggest extensive gene flow in this butterfly across all of Africa and encompassing all regions of the genome outside of the colour pattern loci themselves (Martin et al., 2020). In other words, the interactions between the colour pattern alleles in the contact zone are identical regardless of the source of the parents. This negative result is as striking as the positive nature of the interactions between the colour loci and is consistent with the recent and ongoing nature of the admixture polymorphism in this fascinating butterfly, where dominance has yet to fully evolve. These findings help place the African Monarch system into a unique and ongoing case of dominance evolution. Research priorities are now, therefore, to try and test the fitness of heterozygotes of differing levels of expressivity within the hybrid zone. Such measurements will help us better understand how heterozygote fitness might help maintain the unexpected levels of polymorphism found across East Africa.

## AUTHOR CONTRIBUTIONS

**Richard H. ffrench‐Constant:** Conceptualization (equal); data curation (equal); formal analysis (equal); funding acquisition (equal); investigation (equal); methodology (equal); project administration (equal); writing – original draft (equal); writing – review and editing (equal). **Jonathan Bennie:** Conceptualization (equal); data curation (equal); formal analysis (equal). **Ian J. Gordon:** Conceptualization (equal); data curation (equal); formal analysis (equal); funding acquisition (equal); investigation (equal); methodology (equal). **Lorna Depew:** Data curation (equal); formal analysis (equal); investigation (equal). **David A. S. Smith:** Conceptualization (equal); data curation (equal); formal analysis (equal); investigation (equal); methodology (equal).

## FUNDING INFORMATION

Rff‐C, IG and DAS were supported by a grant from the National Geographic Society. Rff‐C was also supported by a Merit Award from the Royal Society of London and by the Leverhulme Trust under grant number F/00144AY and the Biotechnology and Biological Sciences Research Council under grant number BBE0118451.

## CONFLICT OF INTEREST STATEMENT

The authors declare no conflicting interests.

## Data Availability

R code and raw data can be found at FigShare https://doi.org/10.6084/m9.figshare.22664872.v1.
